# Evaluating the safety and efficiency of day-care hysterectomy: a comparative study using propensity score matching

**DOI:** 10.3389/fmed.2025.1625351

**Published:** 2025-09-05

**Authors:** Qinyan Cao, Tianjiao Liu, Yunyi Su, Xian Wu, Aijie Xie, Hui Wang, Ying Liu, Jie Yu, Tenglan Wu, Xiaoyan Liao, Wei Cheng, Jianmei Liao, Qiang Zhang, Yonghong Lin, Li He, Xiaoqin Gan

**Affiliations:** Chengdu Women’s and Children’s Central Hospital, School of Medicine, University of Electronic Science and Technology of China, Chengdu, China

**Keywords:** hysterectomy, day-care procedure, traditional inpatient procedure, enhanced recovery after surgery, propensity score matching, surgical outcomes

## Abstract

**Objective:**

To compare the outcomes of hysterectomy performed via traditional inpatient procedure versus day-care procedure with a focus on surgical time, post-operative recovery, costs, and patient satisfaction, using propensity score matching (PSM) to adjust for confounding variables.

**Methods:**

A total of 519 patients undergoing hysterectomy were initially identified. After PSM, 340 patients were included for analysis. Primary outcomes included perioperative complications, surgical time, post-operative discomfort, hospital stay, total cost, and patient satisfaction. Multiple linear regression analysis was performed to explore factors associated with operative bleeding and surgical time. Binary logistic regression was employed to analyze the factors influencing postoperative discomfort.

**Results:**

After PSM, the day-care group demonstrated significantly lower hemoglobin decline, post-operative discomfort rates, hospital stay, and total cost, along with higher patient satisfaction (*p* < 0.05). Multivariate analysis showed a significant correlation between post-operative discomfort and both surgical modality and procedure time. For each additional minute of surgery time, the risk of post-operative discomfort increased by 2% (95% CI: 1.01, 1.03, *p* < 0.001). Furthermore, the ERAS-based day-care surgical modality reduced the risk of post-operative discomfort by 80% (95% CI: 0.08, 0.50, *p* < 0.001).

**Conclusion:**

The day-care procedure, guided by an enhanced recovery after surgery protocol, not only reduces hospital stay and overall costs but also improves patient satisfaction and reduces post-operative complications without compromising safety. These findings support the feasibility and benefits of day-care hysterectomy as a viable option for appropriately selected patients, offering significant advantages in terms of recovery and cost-efficiency.

## Background

Hysterectomy is one of the most commonly performed surgical procedures in gynecology, often indicated for conditions such as uterine fibroids, abnormal uterine bleeding, and certain types of gynecological cancers (1, 2). Traditionally, hysterectomies have been conducted as inpatient procedures, requiring hospital admission and an extended recovery period (3, 4). However, advancements in surgical techniques, anesthesia, and perioperative care have facilitated the adoption of minimally invasive approaches, enabling the performance of hysterectomies in a day-care setting (5, 6).

The shift toward day-care procedures aligns with broader trends in healthcare aimed at enhancing cost efficiency, reducing hospital stays, and improving patient satisfaction (7, 8). Day-care hysterectomy offers several potential benefits, including shorter hospital stays, quicker return to daily activities, and reduced healthcare costs. Nonetheless, concerns persist regarding the safety, feasibility, and patient outcomes associated with these procedures, particularly in comparison to traditional inpatient hysterectomies (9).

Previous studies have reported mixed results, with some demonstrating comparable safety and efficacy between the two approaches, while others have raised concerns about potential complications, such as inadequate postoperative pain management and higher rates of readmission (10–12). These inconsistencies underscore the need for robust comparative studies to evaluate the outcomes of traditional inpatient versus day-care hysterectomy procedures.

Propensity score matching (PSM) is a statistical technique increasingly used in observational studies to minimize selection bias and simulate randomized controlled trial conditions (13, 14). By matching patients based on baseline characteristics, PSM allows for a more accurate comparison of outcomes between treatment groups. In this study, we employ PSM to assess and compare the clinical outcomes, cost-effectiveness, and patient-reported satisfaction between traditional inpatient and day-care hysterectomy procedures. In addition, we have provided a detailed standard operating procedure (SOP) process for the day-care surgical management of total hysterectomy based on the enhanced recovery after surgery (ERAS) protocol, serving as a reference for physicians or institutions interested in adopting this procedure.

## Materials and methods

### Study design and participants

This retrospective cohort study was conducted at Chengdu Women’s and Children’s Center Hospital to compare the clinical and economic outcomes of hysterectomies performed via traditional inpatient procedures versus day-care procedures (China Clinical Trial Registration Number: ChiCTR2200059282). The study was approved by the Ethics Committee of Chengdu Women’s and Children’s Center Hospital (Approval No: 2022207). This study was conducted in compliance with the principles of the Declaration of Helsinki. Patient data were anonymized, and confidentiality was ensured throughout the study. As this was a retrospective study, informed consent was waived by the institutional review board.

The study population included women who underwent hysterectomy between April 2020 and April 2024. Inclusion criteria encompassed adult females aged 18 to 65 years with diagnoses indicating elective hysterectomy, such as uterine fibroids, adenomyosis, or precancerous lesions and early-stage malignant tumors. Patients were excluded if they met any of the following criteria: emergency surgeries, severe comorbidities (e.g., ASA score ≥ 4), incomplete follow-up data, concurrent other complex surgical procedures (e.g., lymphadenectomy, cytoreductive surgery, or pelvic reconstructive surgery), multiple elective surgeries, or simultaneous radiation or chemotherapy.

### Data collection

Clinical data were collected from the hospital’s electronic medical records system, including demographic variables such as age, body mass index (BMI), comorbidities, ASA score, and surgical indications. Detailed surgical information was also recorded, encompassing operative time, the type of hysterectomy (transumbilical single-port laparoscopy or multi-port laparoscopy), estimated blood loss, and intraoperative complications. Postoperative data included the length of hospital stay and incidences of postoperative discomfort, such as moderate-to-severe pain, nausea/vomiting, abdominal distension, and significant urinary irritation symptoms. Postoperative complications, including infection, hemorrhage, urinary retention, and thrombosis, were documented along with 30-day readmission rates. Additionally, patient-reported satisfaction was assessed using a 5-point Likert scale during the 6-week postoperative follow-up.

### Day-care procedure management model

#### SOP for day-care surgery admission

1. Outpatient assessment: The gynecologist performs an initial evaluation to determine whether the patient meets the criteria for day-care surgery. The patient undergoes preoperative tests in the outpatient department, and the anesthesiologist assesses whether the patient is suitable for day-care surgery.
2. Surgery scheduling: Patients can schedule the surgery date through multiple channels, such as telephone or WeChat.
3. Reevaluation: Prior to surgery, the patient’s eligibility for day-care surgery is reassessed. After further discussion with the patient, a consent form for surgery is signed, and the procedure is performed.
4. Enhanced recovery strategy: An ERAS strategy is implemented to promote postoperative recovery.
5. Postoperative follow-up: On the morning following surgery, a blood routine test is conducted to monitor the patient’s condition.
6. Discharge evaluation: The patient is discharged if they meet the discharge criteria. The discharge criteria include: no significant abdominal distension, ability to consume liquid food, passage of flatus, smooth spontaneous urination, Post Anesthesia Discharge Scoring System (PADS) score ≥ 9, hemoglobin ≥ 70 g/l, no significant elevation in blood inflammatory markers. Discharge readiness was assessed using the PADS; “mild impairment” corresponded to a score of 1 in a single domain and was defined as: pain NRS ≤ 3 at rest and controlled with oral analgesics; ≤ 1 episode of nausea/vomiting responsive to oral antiemetic; expected surgical oozing not requiring dressing change or hemostasis; and ambulation with minimal assistance without dizziness/syncope. Vital signs had to be stable (BP/HR within ± 20% of baseline and SpO_2_ ≥ 94% on room air), and a total PADS score ≥ 9 was required for discharge.
7. Follow-up management: A telephone follow-up is conducted within 24 h of discharge. On postoperative days 1, 3, 8, and 15, the patient is sent a postoperative follow-up questionnaire via a custom-developed information system. The system collects data on abnormal conditions, and follow-up doctors assess and address any concerns. If necessary, patients are asked to return to the hospital. A satisfaction survey is conducted on day 42 after surgery. The patient is scheduled for follow-up visits at the outpatient clinic 1 week, 1 month, and 3 months postoperatively.

#### Enhanced recovery after surgery protocol

1. Diet, Preoperative: 8 h before surgery (Avoid fried foods, fatty foods, and meats), 6 h before surgery (Avoid starchy solid foods), 2 h before surgery (Drink sugar-containing beverages, but avoid water); Postoperative: After the patient regains consciousness, liquid or soft food can be introduced. Chewing gum is encouraged to promote bowel function recovery.
2. Activity: Postoperatively, once the patient is alert, family members assist the patient in getting out of bed and performing light activities.
3. Catheterization: A urinary catheter is placed during surgery, but it is not retained postoperatively. Patients are encouraged to get out of bed and urinate independently as soon as possible.
4. Thermoregulation: An air blanket is used during surgery to maintain body temperature.
5. Pain Management: 30 min before the surgery ends, 30 mg of ketorolac is administered intravenously for postoperative pain relief. For patients undergoing transumbilical single-port laparoscopic surgery, a local anesthetic (0.30–0.33% ropivacaine) is routinely used for local wound infiltration or ultrasound-guided transversus abdominis plane block. In the recovery room, if the pain score exceeds 3, ibuprofen suspension (10 ml, three times a day) or diclofenac sodium suppositories (50 mg) are given, or tramadol hydrochloride (50 mg) is injected intramuscularly for pain relief. Patient-controlled analgesia pumps are avoided.
6. Antiemetic Management: 30 min before surgery ends, 0.3 mg of ramosetron and 5 mg of dexamethasone are administered intravenously to prevent postoperative nausea and vomiting. For postoperative vomiting, metoclopramide (10 mg) tablets are given orally three times a day or metoclopramide (10 mg) injection intramuscularly.
7. No routine drains: Drain placement is not routinely performed.
8. Minimizing invasive procedures: Preoperatively, there is no routine shaving, no enemas, and no vaginal cleansing. Postoperatively, routine intravenous fluid administration is not performed.

### Traditional inpatient management model

The conventional inpatient management model for total hysterectomy involves a comprehensive perioperative approach. Preoperatively, the patient is admitted one day before surgery, undergoes necessary assessments, and receives preparations such as cleaning the umbilical area, vaginal iodine antiseptic wash, and administration of laxatives or enemas as needed. Fasting is required for 8 h before surgery, and a first-generation cephalosporin is administered for infection prevention 30 min prior to surgery. Intraoperatively, general anesthesia with endotracheal intubation is used, and an abdominal drain, urinary catheter, and patient-controlled analgesia (PCA) pump are placed as needed. Postoperatively, the patient receives fluid resuscitation and infection prevention treatments for 3 days, with gradual reintroduction of food and early ambulation. Abdominal drains and catheters are removed by day 2–3, and venous thromboembolism risk is assessed and managed with appropriate prophylaxis. The patient is typically discharged 4–5 days after surgery, provided they meet discharge criteria.

### Propensity score matching

To reduce selection bias and adjust for confounding factors, PSM was used. A logistic regression model was used to calculate the propensity scores, incorporating key baseline characteristics that could influence the decision to undergo either inpatient or day-care hysterectomy. These variables included age, BMI, smoking history, alcohol consumption history, diabetes, hypertension, history of pelvic surgery, surgical indications, surgical approach, and uterine weight. PSM was conducted using 1:1 nearest-neighbor matching with a caliper width equal to 0.2 of the standard deviation of the logit of the propensity score, as recommended to minimize bias and mean squared error. Only patients with a matched pair were included in the final analysis, ensuring a balanced comparison between the two groups. All hysterectomies were performed by the same senior surgical team under a standardized ERAS pathway; therefore, the individual surgeon was not included as a covariate in the PSM.

PSM was performed to achieve covariate balance (standardized mean difference ≤ 0.10) rather than exact matching on category counts; consequently, small differences in the numbers within surgical-indication strata may persist after matching.

### Outcome measures

The primary outcomes of the study were:

Length of hospital stay: Measured in days, defined as the time from the completion of surgery to hospital discharge.

Postoperative complications: Including, but not limited to, surgical site infection, bleeding, thromboembolic events, and urinary complications. The grading of complications was based on the modified Clavien-Dindo classification system, as adapted by our hospital (Supplementary Table 1).

30-day readmission rates: The proportion of patients who were readmitted to the hospital within 30 days of discharge due to any complication.

The secondary outcomes included:

Total procedural cost: Evaluated from a healthcare provider perspective, encompassing direct costs such as surgery, anesthesia, hospitalization, and postoperative care. To ensure comparability, we defined “total procedural cost” as the episode-of-care cost from preoperative evaluation through discharge. For day-care patients, this included outpatient preoperative tests and the anesthesiologist assessment (e.g., laboratory tests, imaging, anesthesia clinic), which in Sichuan Province are billed to and reimbursed by the provincial medical insurance as part of the surgical encounter and were therefore captured in our cost dataset. Analogous preoperative items for inpatients were also included.

Patient satisfaction: Measured using a 5-point Likert scale (ranging from very dissatisfied to very satisfied) based on their overall experience with the procedure and the recovery process.

### Statistical analysis

Baseline characteristics of patients in the two groups (inpatient and day-care) were compared both before and after propensity score matching. Descriptive statistics were used to summarize categorical variables (e.g., frequency and percentages) and continuous variables (mean and standard deviation). Paired *t*-tests were conducted for normally distributed continuous variables, while the Kruskal–Wallis test was applied for non-normally distributed continuous variables. The chi-squared test and Fisher’s exact test were used for comparisons of paired categorical variables. Multiple linear regression analysis was performed to explore factors associated with operative bleeding and surgical time. Binary logistic regression was employed to analyze the factors influencing postoperative discomfort. All tests were two-tailed, and a *p*-value of < 0.05 was considered statistically significant.

## Results

**FIGURE 1:**
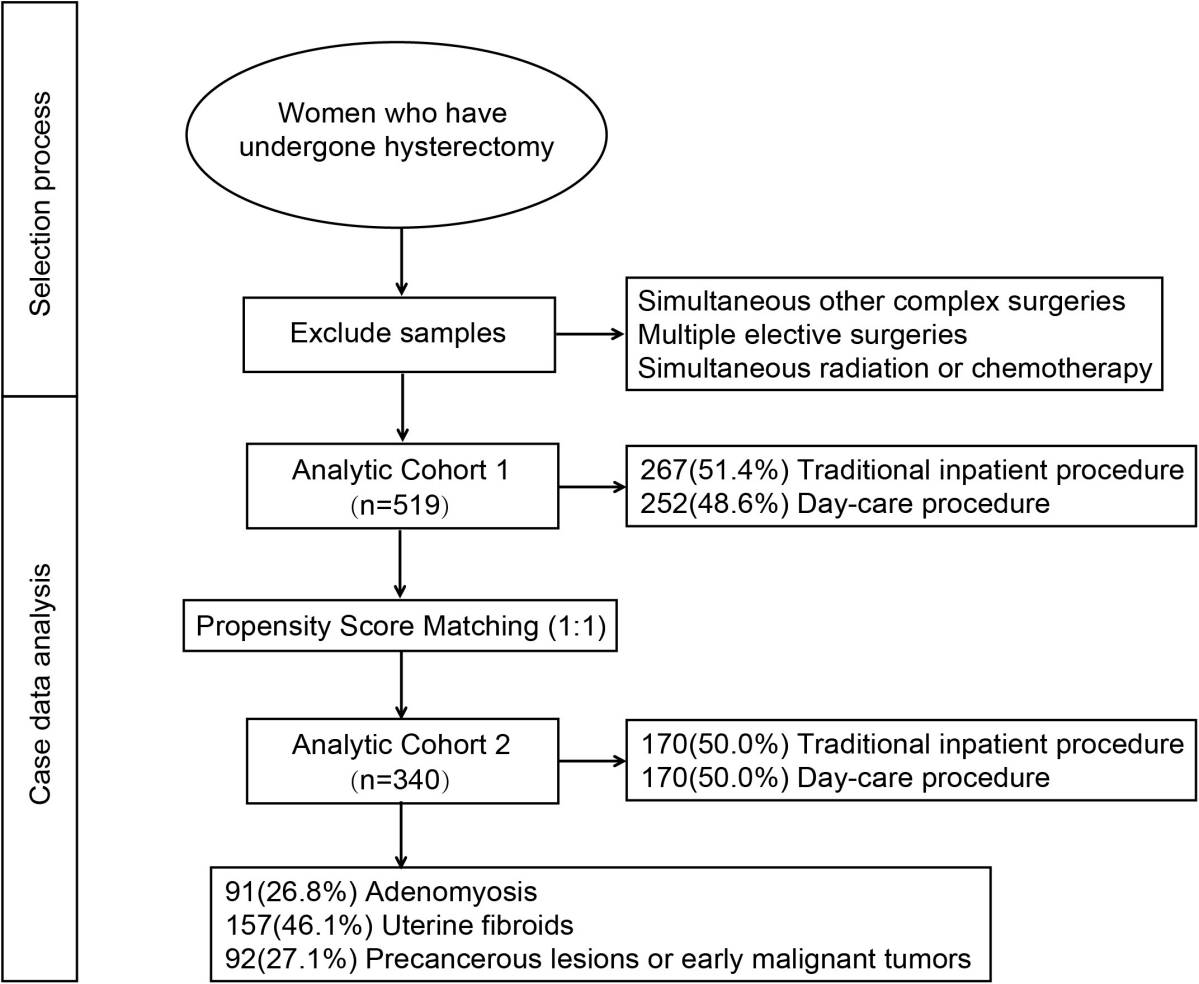
The selection process for this study.

The patient recruitment process for this study is depicted in Figure 1. After excluding cases with concurrent complex surgical procedures, multiple elective surgeries, or simultaneous radiation or chemotherapy, a total of 519 patients were initially available for analysis. Following PSM based on factors such as age, BMI, smoking history, alcohol consumption history, diabetes, hypertension, history of pelvic surgery, surgical indications, surgical approach, and uterine weight, the final analysis included 340 patients. Patients’ demographic data are shown in Table 1. After PSM, the average age was 49.44 ± 4.97 years, and the mean BMI was 23.96 ± 3.02 kg/m^2^. Furthermore, 44.8% of patients had a history of pelvic surgery. Regarding the indications for total hysterectomy, 91 patients (26.8%) underwent surgery for adenomyosis, 157 patients (46.1%) for uterine fibroids, and 92 patients (27.1%) for precancerous lesions or early malignant tumors.

**TABLE 1:** Description of the patients demographic characteristics and surgical modality.

Variables	Before PSM	After PSM
Patients	519	340
Age (year)	49.32 ± 5.22	49.44 ± 4.97
BMI (kg/m^2^)	23.97 ± 3.01	23.96 ± 3.02
Smoking	10 (1.9%)	0 (0%)
Alcohol consumption history	5 (0.9%)	0 (0%)
Diabetes	18 (3.4%)	12 (3.5%)
Hypertension	97 (18.6%)	58 (17.0%)
History of pelvic surgery	233 (44.9%)	152 (44.8%)
**Indications for surgery**		
Adenomyosis	138 (26.6%)	91 (26.8%)
Uterine fibroids	230 (44.3%)	157 (46.1%)
Precancerous lesions or early malignant tumors	151 (29.1%)	92 (27.1%)
**Surgical approach**		
Transumbilical single-port laparoscopy	102 (19.6%)	67 (19.7%)
Multi-port laparoscopy	417 (80.4%)	273 (80.3%)
**Surgical modality**		
Traditional inpatient procedure	267 (51.4%)	170 (50.0%)
Day-care procedure	252 (48.6%)	170 (50.0%)

PSM, propensity score matching.

Supplementary Figure 1 illustrates the changes in the standardized mean difference (SMD) for each variable before and after PSM. Before matching, the SMD values were large, indicating significant differences between the two groups. After PSM, all SMD values were < 0.1, except for the history of pelvic surgery, suggesting a balanced distribution of variables between the two groups. The post-matching clinical characteristics of the groups showed a statistically balanced distribution, effectively eliminating selection bias. After PSM, the day-care surgery group showed a lower decline in hemoglobin levels, a reduced rate of post-operative discomfort, shorter hospital stay, and lower total cost, while also exhibiting higher patient satisfaction (*P* < 0.05) (Table 2).

**TABLE 2:** Description of the patient characteristics by surgical modality.

	Before PSM			After PSM		
Variables	Inpatient surgery *N* = 267	Day-care procedure *N* = 252	*P*	Inpatient procedure *N* = 170	Day-care procedure *N* = 170	*P*
Age (year)	49.25 ± 5.38	49.40 ± 5.05	0.757^a^	49.42 ± 5.21	49.47 ± 4.74	0.922^a^
BMI (kg/m^2^)	24.08 ± 3.13	23.86 ± 2.87	0.415^a^	23.84 ± 3.13	24.08 ± 2.91	0.451^a^
Smoking	3 (1.1%)	7 (2.8%)	0.211^c^	0 (0%)	0 (0%)	–
Alcohol consumption history	2 (0.7%)	3 (1.2%)	0.678^c^	0 (0%)	0 (0%)	–
Diabetes	8 (3.0%)	10 (4.0%)	0.545^b^	6 (3.5%)	6 (3.5%)	1.000^b^
Hypertension	55 (20.6%)	42 (16.7%)	0.251^b^	29 (17.1%)	29 (17.1%)	1.000^b^
History of pelvic surgery	133 (49.8%)	100 (39.7%)	0.062^b^	81 (47.6%)	71 (41.8%)	0.418^b^
Indications for surgery			0.128^b^			0.970^b^
Adenomyosis	71 (26.6%)	67 (26.6%)		45 (26.5%)	46 (27.1%)	
Uterine fibroids	128 (47.9%)	102 (40.5%)		78 (45.9%)	79 (46.5%)	
Precancerous lesions or early malignant tumors	68 (25.5%)	83 (32.9%)		47 (27.6%)	45 (26.5%)	
Surgical approach			0.152^b^			1.000^b^
Single-port	46 (17.2%)	56 (22.2%)		34 (20.0%)	33 (19.4%)	
Multi-port	221 (82.8%)	196 (77.8%)		136 (80.0%)	137 (80.6%)	
**Operative information**						
Procedure time (min)	140.87 ± 55.52	131.49 ± 40.36	0.028^d^	136.24 ± 51.26	130.54 ± 40.69	0.257^d^
Bleeding volume (ml)	85.24 ± 106.46	74.01 ± 106.24	0.230^a^	80.41 ± 89.95	70.35 ± 98.18	0.325^a^
Uterine weight (g)	375.21 ± 216.83	341.98 ± 176.25	0.085^a^	323.55 ± 136.63	318.02 ± 146.36	0.802^a^
**Post-operative information**						
Hemoglobin decline (g/L)	16.61 ± 9.22	12.58 ± 8.59	< 0.001^a^	16.55 ± 9.45	12.22 ± 8.73	< 0.001^a^
Discomfort	73 (27.3%)	33 (13.1%)	< 0.001^b^	52 (30.6%)	20 (11.8%)	< 0.001^b^
Pain	28 (10.5%)	18 (7.1%)	0.180^b^	20 (11.8%)	11 (6.5%)	0.090^b^
Nausea	12 (4.5%)	4 (1.6%)	0.074^c^	8 (4.7%)	2 (1.2%)	0.104^c^
Distension	16 (6.0%)	6 (2.4%)	0.041^b^	12 (7.1%)	4 (2.4%)	0.070^c^
Urinary irritation	17 (6.4%)	5 (2.0%)	0.013^b^	12 (7.1%)	3 (1.8%)	0.031^c^
Unplanned re-surgery	5 (1.9%)	3 (1.2%)	0.725^c^	3 (1.8%)	3 (1.8%)	1.000^c^
Perioperative complications	5 (1.9%)	4 (1.6%)	1.000^c^	2 (1.2%)	1 (0.6%)	1.000^c^
Hospital stay	5.94 ± 1.75	1.34 ± 0.71	< 0.001^d^	6.00 ± 1.88	1.35 ± 0.68	< 0.001^d^
Total cost (thousand RMB)	16.73 ± 3.20	9.83 ± 1.68	< 0.001^d^	16.75 ± 3.53	9.93 ± 1.73	< 0.001^d^
Unplanned readmissions	8 (3.0%)	5 (2.0%)	0.578^c^	5 (2.9%)	4 (2.4%)	1.000^c^
Patient satisfaction	247 (92.5%)	249 (98.8%)	< 0.001^c^	159 (93.5%)	167 (98.2%)	0.029^c^

PSM, propensity score matching; BMI, body mass index.

^a^Average and standard deviation. One-way analysis of variance.

^b^Number (percentage). Chi-squared Test.

^c^Number (percentage). Fisher exact test.

^d^Average and standard deviation. Kruskal–Wallis test.

After PSM, the inpatient procedure group included 45 cases of adenomyosis, 78 cases of uterine fibroids, and 47 cases of precancerous lesions or early malignant tumors, whereas the day-care procedure group included 46, 78, and 45 cases, respectively; the between-group difference was not significant (*p* = 0.970). After PSM, 52 patients (30.6%) in the inpatient procedure group and 20 patients (11.8%) in the day-care procedure group reported postoperative discomfort. Among them, pain was the most frequent symptom (20 [11.8%] vs. 11 [6.5%]), followed by urinary irritation (12 [7.1%] vs. 3 [1.8%]), distension (12 [7.1%] vs. 4 [2.4%]), and nausea (8 [4.7%] vs. 2 [1.2%]). These results suggest that the day-care procedure, under an ERAS protocol, not only reduces the overall incidence of discomfort but also particularly decreases urinary irritation (*P* < 0.05) (Table 2).

**FIGURE 2:**
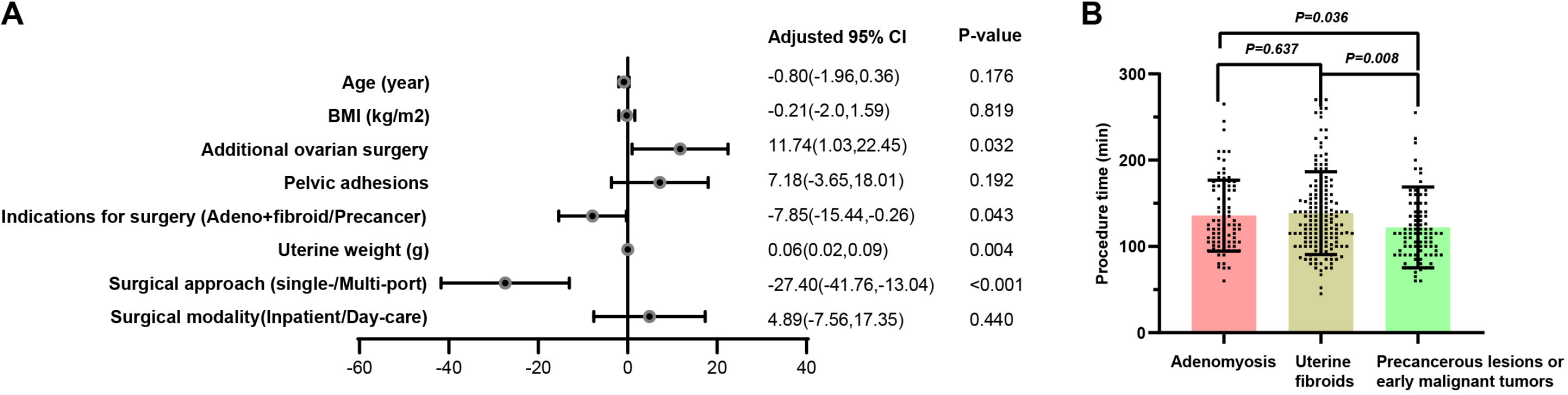
The impact of perioperative characteristics on procedure time. **(A)** After adjusting for variables such as age, BMI, pelvic adhesions, and surgical modality, the results revealed a significant correlation between procedure time and additional ovarian surgery (11.74, 95% CI: 1.03, 22.45, *p* = 0.032), indications for surgery (–7.85, 95% CI: –15.44 to –0.26, *p* = 0.043), uterine weight (0.06, 95% CI: 0.02, 0.09, *p* = 0.004), and surgical approach (–27.40, 95% CI: –41.76 to –13.04, *p* < 0.001). With each 100 g increase in uterine weight, the duration of surgery increased by approximately 6 min. Performing concurrent ovarian surgery increased the surgery duration by about 11.7 min, while multi-port laparoscopy reduced the procedure time by 27.3 min; **(B)** Further analysis was conducted to compare the average surgery duration among patients with three different surgical indications. The results showed that patients undergoing surgery for precancerous lesions or early malignant tumors had a significantly shorter surgery time compared to those undergoing surgery for adenomyosis and uterine fibroids (Adenomyosis vs. precancer, *p* = 0.036; Uterine fibroids vs. precancer, *p* = 0.0008).

Factors influencing the duration of surgery were further explored using multivariate linear regression analysis (Figure 2A). After adjusting for variables such as age, BMI, pelvic adhesions, and surgical modality, the results revealed a significant correlation between procedure time and additional ovarian surgery (11.74, 95% CI: 1.03, 22.45, *p* = 0.032), indications for surgery (−7.85, 95% CI: −15.44 to −0.26, *p* = 0.043), uterine weight (0.06, 95% CI: 0.02, 0.09, *p* = 0.004), and surgical approach (−27.40, 95% CI: −41.76 to −13.04, *p* < 0.001). With each 100 g increase in uterine weight, the duration of surgery increased by approximately 6 min. Performing concurrent ovarian surgery increased the surgery duration by about 11.7 min, while multi-port laparoscopy reduced the procedure time by 27.3 min Further analysis was conducted to compare the average surgery duration among patients with three different surgical indications. The results showed that patients undergoing surgery for precancerous lesions or early malignant tumors had a significantly shorter surgery time compared to those undergoing surgery for adenomyosis and uterine fibroids (Adenomyosis vs. precancer, *p* = 0.036; Uterine fibroids vs. precancer, *p* = 0.0008) (Figure 2B).

Binary logistic regression analysis was used to further investigate the influencing factors of post-operative discomfort (Table 3). After adjusting for variables such as age, BMI, history of pelvic surgery, indications for surgery, uterine weight, bleeding volume, surgical approach, and hemoglobin decline, the results revealed a significant correlation between post-operative discomfort and both surgical modality and procedure time. For each additional minute of surgery time, the risk of post-operative discomfort increased by 2% (95% CI: 1.01, 1.03, *p* < 0.001). Furthermore, the ERAS-based day-care surgical modality reduced the risk of post-operative discomfort by 80% (95% CI: 0.08, 0.50, *p* < 0.001).

**TABLE 3:** Association between post-operative discomfort and perioperative characteristics.

Variables	Exp (B)	95% CI	*P*-value
Surgical modality (inpatient/day-care)	0.20	(0.08, 0.50)	< 0.001
Age (year)	1.03	(0.94, 1.14)	0.491
BMI (g/m^2^)	0.98	(0.84, 1.14)	0.750
History of pelvic surgery	0.82	(0.42, 1.60)	0.560
Indications for surgery	1.60	(0.85, 3.02)	0.144
Uterine weight (g)	1.00	(0.99, 1.01)	0.161
Bleeding volume (ml)	1.00	(0.99, 1.01)	0.574
Procedure time (min)	1.02	(1.01, 1.03)	< 0.001
Surgical approach	0.65	(0.21, 1.98)	0.443
Hemoglobin decline (g/L)	0.99	(0.94, 1.03)	0.591

Multivariate linear regression analysis was used to further explore the influencing factors of operative bleeding. After adjusting for variables such as age, BMI, additional ovarian surgery, pelvic adhesions, indications for surgery, uterine weight, surgical approach, and surgical modality, the results revealed a significant correlation between operative bleeding and procedure time. For each additional minute of surgery, operative bleeding increased by 1.27 ml (95% CI: 1.01, 1.53, *p* < 0.001) (Table 4).

**TABLE 4:** Association between operative bleeding and perioperative characteristics.

Variables	Beta	95% CI	*P*-value	VIF
***R*^2^ = 0.329**				
Age (year)	−0.05	(−2.34, 2.23)	0.963	1.25
BMI (g/m^2^)	−2.29	(−5.82, 1.24)	0.203	1.02
Additional ovarian surgery	−10.84	(−32.13, 10.46)	0.317	1.21
Pelvic adhesions	−16.94	(−38.33, 4.45)	0.120	1.19
Indications for surgery	0.82	(−14.25, 15.89)	0.914	1.24
Uterine weight (g)	−0.01	(−0.09, 0.06)	0.736	1.49
Surgical approach	4.76	(−24.40, 33.92)	0.748	1.21
Surgical modality (inpatient/day-care)	−2.18	(−26.73, 22.36)	0.861	1.22
Procedure time	1.27	(1.01, 1.53)	< 0.001	1.22

## Discussion

This study aimed to compare the clinical and economic outcomes of hysterectomy performed via traditional inpatient procedure and day-care procedure, utilizing PSM to reduce selection bias. The results demonstrated that the day-care procedure was associated with several advantages over the traditional inpatient procedure, including shorter hospital stays, lower postoperative discomfort, reduced total cost, and higher patient satisfaction. These findings underscore the potential benefits of adopting a day-care approach in selected patients undergoing elective hysterectomy.

One of the most significant findings of this study was the reduced postoperative discomfort observed in the day-care group. This is consistent with previous studies that have shown that day-care surgery, which typically involves enhanced recovery protocols and early mobilization, can lead to reduced postoperative pain and discomfort (15, 16). The implementation of an ERAS protocol in the day-care group likely contributed to the faster recovery and lower incidence of postoperative complications. Specifically, the use of multimodal analgesia, early mobilization, and avoidance of prolonged catheterization in the day-care group may have facilitated improved recovery outcomes. The association between procedure time and postoperative discomfort also warrants attention; each additional minute of surgery time was found to increase the risk of post-operative discomfort by 2%. This highlights the importance of optimizing surgical efficiency in order to minimize patient discomfort and enhance recovery outcomes.

In terms of hospital stay, the day-care group demonstrated a significantly shorter length of stay compared to the traditional inpatient group. This aligns with the objectives of the ERAS protocol, which emphasizes early mobilization, minimal invasive interventions, and expedited discharge. These strategies contribute to a reduction in hospital stay without compromising patient safety or clinical outcomes (17, 18). Notably, the total cost associated with the day-care procedure was also lower, likely attributed to the reduced length of stay and fewer postoperative resources required for care. By shortening hospital stays and decreasing hospitalization costs, day-care surgery not only alleviates the financial burden on patients and healthcare insurance but also reduces overall healthcare costs (19, 20). This approach benefits patients, insurance providers, and hospitals alike, making it a model that deserves widespread adoption and further promotion.

We developed an ERAS-based day-care surgery SOP for our practice. A multimodal analgesic approach was used to manage postoperative pain, and compared to patients who traditionally used patient-controlled analgesia, the incidence of moderate to severe pain did not increase. Interestingly, we observed a significant reduction in the rate of non-infectious postoperative fever. This could be attributed to the routine administration of NSAIDs, which have antipyretic effects (21, 22). While the slight increase in body temperature due to heat absorption postoperatively did not have any adverse effects on the patients, in China, postoperative fever often leads to unnecessary antibiotic use. Reducing postoperative fever may help lower the usage of antibiotics and prevent prolonged hospital stays.

In our cases, we did not place pelvic drainage tubes or urinary catheters postoperatively. With adequate pain control and psychological preparation, patients were encouraged to get out of bed and urinate independently at an early stage. Only 3.03% of patients experienced urinary symptoms, and 0.51% had urinary retention, with no cases of urinary tract infections. This approach did not increase the incidence of urinary symptoms, urinary retention, or infections compared to patients who had urinary catheters retained for 2–3 days. This suggests that not retaining urinary catheters after laparoscopic total hysterectomy under general anesthesia is feasible, which is consistent with previous studies (23, 24).

In this study, multi-port laparoscopy demonstrated a distinct advantage in reducing surgical time compared to single-port umbilical laparoscopy. This is likely due to the greater flexibility and maneuverability provided by the additional ports, allowing for more efficient instrument placement and better visualization of the surgical field (25, 26). Multi-port laparoscopy facilitates the use of specialized instruments, enabling quicker access to and manipulation of the uterus and surrounding structures. In contrast, single-port umbilical laparoscopy, while minimally invasive, requires more complex instrument maneuvering through a single port, which can limit the range of motion and complicate the procedure, particularly in cases involving larger uteri or complex pathologies.

Although propensity score matching effectively balanced most baseline characteristics, the variable “history of pelvic surgery” remained slightly imbalanced (SMD > 0.1). This residual imbalance may have introduced confounding, as prior pelvic surgery could influence intraoperative adhesions, operative difficulty, or postoperative recovery. However, in our multivariate regression models, “history of pelvic surgery” was not significantly associated with postoperative discomfort or operative bleeding, suggesting that its impact on the main outcomes of this study might be limited. Nevertheless, this factor should be interpreted with caution, and future prospective studies with stricter control of surgical history are warranted to validate our findings.

Our study also revealed that the satisfaction level of patients in the day-care procedure group was significantly higher than that of the traditional inpatient group. This improvement in satisfaction may be attributed to the implementation of the ERAS protocol, which reduced invasive procedures such as bowel preparation, intravenous fluid administration, urinary catheterization, and abdominal drainage tubes. These measures likely enhanced patient comfort. Additionally, the reduction in hospital costs and the alleviation of the financial burden on patients may have contributed to the higher satisfaction in the day-care group. This is consistent with previous research. A randomized controlled trial conducted in Italy showed that the implementation of the ERAS protocol in laparoscopic hysterectomy significantly reduced hospital stay without increasing postoperative complication rates (27). Similarly, a study from Canada demonstrated that the application of ERAS strategies in minimally invasive hysterectomy enhanced patient recovery and substantially lowered postoperative hospitalization costs (28). Furthermore, several other studies have also confirmed the effectiveness of ERAS in total hysterectomy procedures (29, 30). These findings collectively underscore the benefits of ERAS in improving both clinical outcomes and economic efficiency in hysterectomy surgeries.

Despite the promising findings, this study has several limitations. As a single-center retrospective cohort study, selection bias and residual confounding cannot be completely excluded, even after PSM, particularly regarding unmeasured factors such as socioeconomic status and health literacy. The sample size, although relatively large, may not fully capture patient heterogeneity, and surgeon decision-making may have led to more straightforward cases being allocated to the day-care setting. In addition, patient satisfaction was assessed only as a binary outcome, limiting the ability to capture gradations of patient experience, and follow-up was restricted to 42 days, precluding evaluation of late complications or long-term recovery. Furthermore, while our results suggest cost advantages of day-care hysterectomy, broader cost-effectiveness across different healthcare systems remains to be explored. Future multicenter randomized trials with extended follow-up and more detailed patient-reported outcomes are needed to validate and generalize these findings.

## Conclusion

In conclusion, this propensity score-matched study suggests that the day-care procedure for hysterectomy offers several advantages over the traditional inpatient procedure, including reduced postoperative discomfort, shorter hospital stays, lower costs, and improved patient satisfaction. Factors such as surgery time, surgical approach, and surgical indication significantly influence operative outcomes, underscoring the importance of individualized patient management in surgical decision-making. The findings of this study support the implementation of day-care hysterectomy in appropriately selected patients as part of an enhanced recovery strategy, with the potential for improving both clinical and economic outcomes. Further prospective studies are needed to validate these results and refine patient selection criteria for day-care procedures.

## Data Availability

The original contributions presented in this study are included in this article/Supplementary material, further inquiries can be directed to the corresponding authors.

## Supplementary material

Supplementary Figure 1 The standardized mean difference (SMD illustrates the changes for each variable before and after PSM). Before matching, the SMD values were large, indicating significant differences between the two groups. After PSM, all SMD values were < 0.1, except for the history of pelvic surgery, suggesting a balanced distribution of variables between the two groups.
