# Supplementary figures and images for: Evaluating the safety and efficiency of day-care hysterectomy: a comparative study using propensity score matching
Source: Front Med (Lausanne). 2025 Sep 5;12:1625351. doi: 10.3389/fmed.2025.1625351 (PMC12446359; doi:10.3389/fmed.2025.1625351)

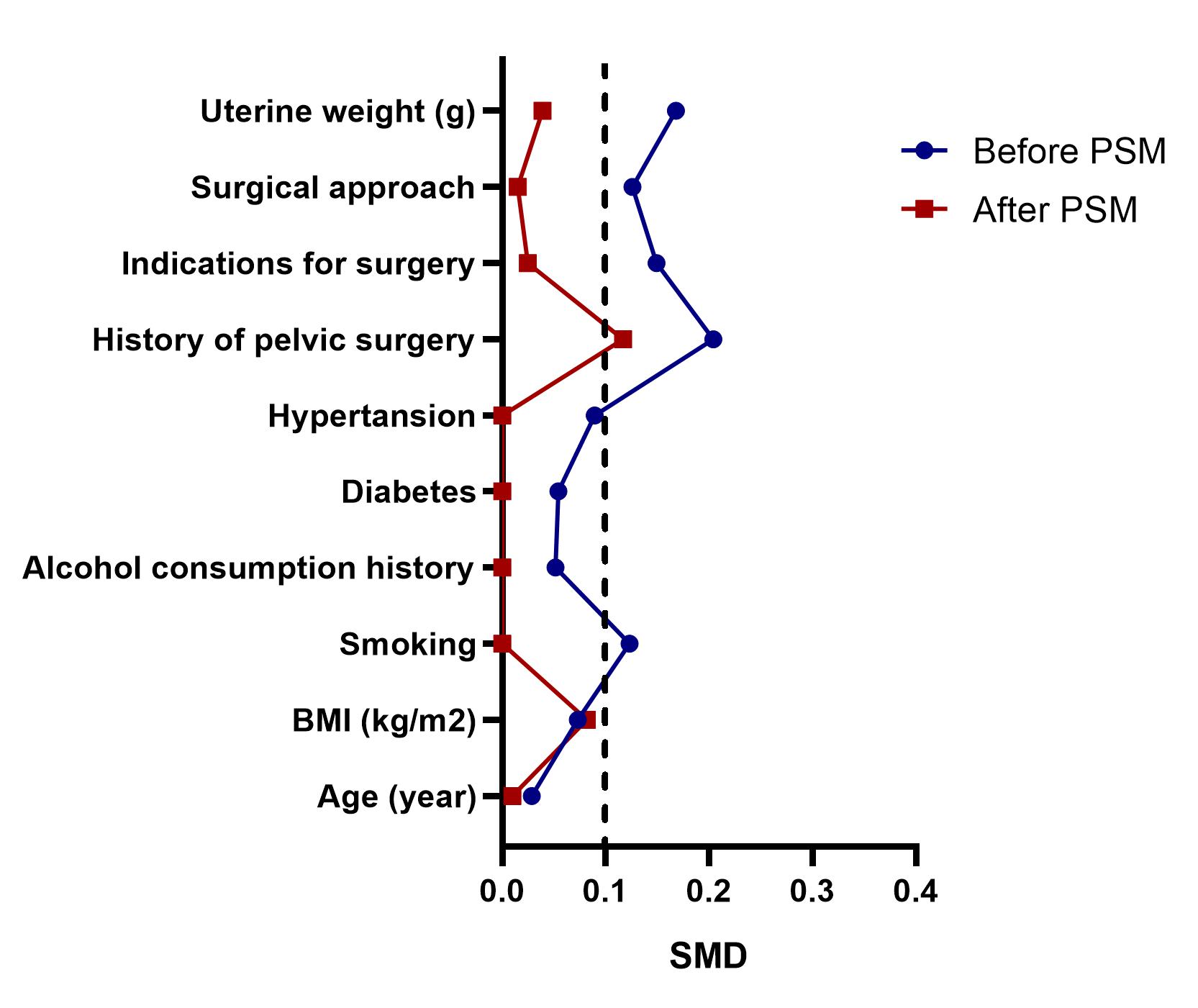

Supplement: Supplementary Figure 1 — The standardized mean difference (SMD illustrates the changes for each variable before and after PSM). Before matching, the SMD values were large, indicating significant differences between the two groups. After PSM, all SMD values were < 0.1, except for the history of pelvic surgery, suggesting a balanced distribution of variables between the two groups. [file Image_1.JPEG]
